# Effects of Far Infrared Radiation and Sitz Bath on Perineal Wound Healing and Pain in Primiparous Women Undergoing an Episiotomy: A Randomized Prospective Parallel Arm Study

**DOI:** 10.7759/cureus.67477

**Published:** 2024-08-22

**Authors:** Srushti Ramesh, Saswati Tripathy, Maitrayee Sen, Dhruva Nandi

**Affiliations:** 1 Obstetrics and Gynaecology, SRM Medical College Hospital and Research Centre, Kattankulathur, IND; 2 Medical Research (Public Health), SRM Medical College Hospital and Research Centre, Kattankulathur, IND

**Keywords:** perineal wound healing, perineal pain, sitz bath, far-infrared radiation, episiotomy

## Abstract

Introduction

Episiotomy is a common surgical procedure done during childbirth. The study aims to assess the efficacy of far-infrared (FIR) and sitz bath (SB) effects on pain relief and healing of perineal wounds in primiparous women who had undergone an episiotomy.

Materials and methods

A randomized prospective parallel arm study was conducted among 208 primigravida women who underwent episiotomy in a tertiary hospital in the southern part of India from December 2020 to March 2022. Participants were randomized into the FIR (n-104) and SB therapy groups (n-104) and their efficacy was assessed for pain relief and healing of perineal wounds using the Visual Analog Scale (VAS), Redness, Oedema, Ecchymosis, and Discharge, the Approximation Scale (REEDA) and the Modified Oxford Scale (MOS). The variables were compared using an independent two-sample t-test and chi-square test (p ≤ 0.05).

Results

Pain was evaluated using VAS, and by the sixth week postpartum, 90.4% (94) of the FIR group reported mild to no pain compared to 88.5% (92) of the SB group. Wound integrity assessment using the REEDA scale showed better results among the FIR group (94, 90.4%) than the SB group (93, 89.4%) on the second day postpartum. Perineal muscle tone, measured by the MOS, was slightly better in the FIR group (59, 62.8%) than the SB group (55, 59.8%) at the sixth week postpartum, although these differences were not statistically significant.

Conclusion

FIR therapy reduces postpartum discomfort, promotes wound healing, and improves perineal muscle tone better than SB therapy. FIR also enhanced patient compliance and efficacy.

## Introduction

An episiotomy is a surgical incision performed over the female perineum just before the head of the fetus crowns to increase the diameter of the pelvic outlet, thus smoothening the delivery process. Episiotomy is one of the most often-used surgical procedures during childbirth. As per a randomized clinical trial done in Brazil, every third of women who gave birth vaginally had undergone episiotomy [1]. Globally, the incidence of episiotomy ranged from 20% to 62.5% [2]. Episiotomy is often performed in an effort to prevent the tearing of soft tissues, which may start with the perineal skin and progress into the perineal muscles and the sphincter ani. Episiotomy should be used for various indications, like shoulder dystocia/difficult shoulder delivery, breech delivery, fetal macrosomia, operative vaginal deliveries like forceps or vacuum-assisted delivery, and several other situations where avoiding an episiotomy can cause serious perineal rupture or laceration.

Episiotomy-related pain and discomfort not only hinder mother-infant contact and breastfeeding [3] but also affect the subsequent emotional healing of the mother. The perineum is a very sensitive and highly vascular area, and episiotomy sutures can result in significant discomfort and anguish. Different techniques have been developed to assess the degree of pain, wound integrity, and perineal muscle tone following an episiotomy. The Visual Analogue Scale (VAS) [4], Numerical Pain Scale (NPS) [5], etc., are used for pain assessment; the Redness, Oedema, Ecchymosis, Discharge, and Approximation Scale (REEDA) [6] is used for wound integrity; and the Modified Oxford Scale (MOS) [7] as well as electromyography are used for assessing the perineal tone. Various methods have been used in aiding the healing process, which include the use of analgesics, ice packs, or ice cubes; maintaining the episiotomy site and the adjacent area clean; avoiding applying pressure to the sutures; using a sitz bath (SB); performing the kegel exercise; and using dry heat topically [8]. Far-infrared radiation (FIR) promotes pain relief and an increase in blood flow, which not only boosts the tissues' availability of oxygen and nutrients but also speeds up the clearance of waste products and aids in the reduction of inflammation. An SB may be helpful for patients who underwent an episiotomy during childbirth, had uncomfortable hemorrhoids, or had perineal irritation by increasing circulation, lessening inflammation, as well as relaxing the muscles. Hence, in our study, we have attempted to assess the efficacy of FIR and the SB effect on the pain relief and healing of perineal wounds in primiparous women who underwent an episiotomy.

## Materials and methods

Study participants

This was an interventional, parallel-arm, randomized prospective study conducted in the obstetrics and gynecology department at a tertiary healthcare center in the southern part of India from December 2020 to March 2022. A total of 245 primigravidae women were screened, out of whom 208 agreed to participate in the study. Proper written informed consent was obtained after giving detailed information about the survey to each patient before enrolling in the study. The inclusion criteria were primiparous women of term gestation (37-42 weeks of gestation) within the age group of 18-35 years, delivered vaginally with episiotomy, willing to participate in the study, and of Indian origin. Exclusion criteria were multiparous women who had a 3rd or 4th-degree perineal tear, underwent instrumental deliveries such as forceps or vacuum-assisted deliveries, and had macrosomic babies (birth weight > 4 kg). This research was approved by the Institutional Ethics Clearance Subcommittee (3015/IEC/2020).

Study procedure

**Figure 1:**
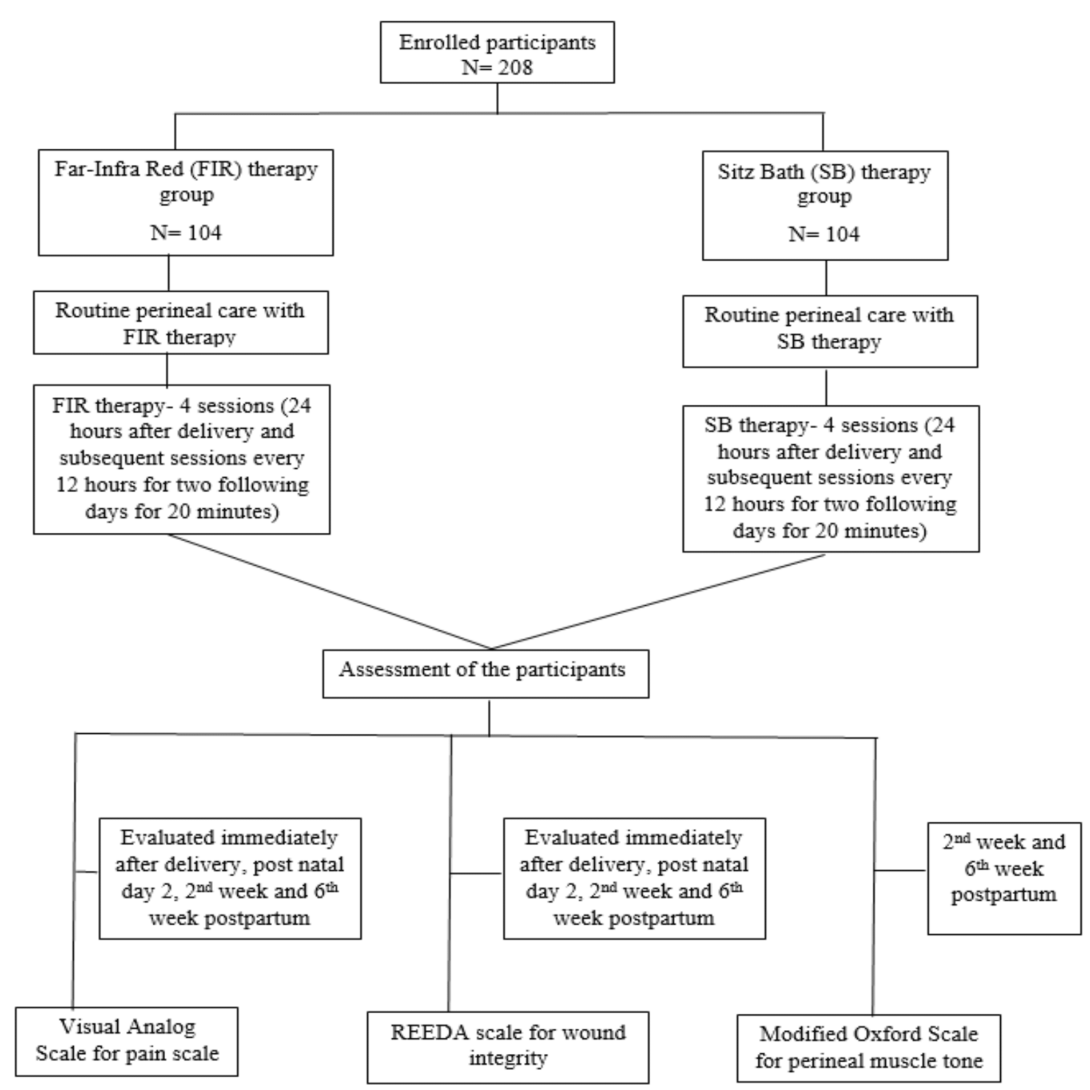
Structural flowchart depicting the methodology of the study This figure illustrates the structural flow of the methodology adopted in this study for the administration of FIR and SB therapy along with the assessment of the participants based on pain, wound integrity, and tone of perineal muscle.

The enrolled participants were randomized into two groups: the FIR group (104 participants) and the SB group (104 participants) (Figure 1). Both groups received routine perineal care, in addition to which the FIR group was given infrared therapy and the SB group was given SB therapy. The envelope method of randomization was used for the collection of data. Both the FIR treatment and SB therapy were scheduled for four sessions: the first one was within 24 hours of delivery, and the subsequent sessions were every 12 hours for the following two days (Figure 1). In the FIR group, women were told to lie down in a supine position with their knees bent and their perineum exposed. For the next 20 minutes, far-FIR radiation treatment using a Murphy FIR lamp (150 watts) was administered from a distance of 30 cm. The SB group was told to sit in the SB containing lukewarm water for over 20 minutes; the patient was assessed carefully regarding any discomfort or difficulty during the process. During the therapy or after completion of the therapy, if the participants had any discomfort or sensitivity, those who refused subsequent sessions were excluded from the study.

Study tools

The scoring for pain was evaluated using VAS, wound integrity was assessed using the REEDA score, and the tones of the perineal muscles were assessed using MOS.

Tool I: Visual Analogue Scale (VAS)

VAS is a tool to assess pain severity [9]. It is a self-reported scale with a horizontal line used to estimate a patient's subjective discomfort. It comprises a 10-point numerical scale corresponding to the degree of pain, with zero indicating no discomfort and 10 signifying the most severe agony. Scores 1, 2, and 3 indicate mild pain; scores 4, 5, and 6 indicate moderate pain; and scores 7, 8, and 9 indicate severe pain. A score of 10 represents the most intolerable suffering.

Tool II: Redness, Oedema, Ecchymosis, Discharge, Approximation Scale (REEDA)

The REEDA scale was created by Davidson [10]. It is an observational checklist to assess episiotomy wound healing. It may be used to assess any kind of postpartum perineal injury. It consists of five components: redness, edema, ecchymosis, discharge, and wound edge approximation. Each component is assigned a score from 0 to 3, as follows: redness scale: 0 (none), 1 (mild within 0.25 cm of incision), 2 (moderate within 0.5 cm of incision bilaterally), and 3 (severe more than 0.5 cm). Edema: 0 = none; 1 = mild perineal, less than 1 cm from the incision; 2 = moderate perineal and/or vulvar, 1-2 cm from the incision; and 3 = severe perineal and/or vulvar, more than 2 cm from the incision. Ecchymosis is graded as 0 = none, 1 = mild within 0.25 cm bilaterally or 0.5 cm unilaterally, 2 = moderate between 0.25 and 1 cm bilaterally or between 0.5 and 2 cm unilaterally, and 3 = severe greater than 1 cm bilaterally or 2 cm unilaterally. Discharge is graded as 0 = none, 1 = serous, 2 = serosanguinous, and 3 = bloody and purulent. The approximation is graded as follows: 0 = closed; 1 = mild skin separation of 3 mm or less; 2 = moderate skin and subcutaneous fat separation; and 3 = severe skin, subcutaneous fat, and fascial layer separation.

The REEDA score ranges from 0 to 15. A high score signals poor wound healing, while a low score indicates great wound healing. The total score on the REEDA scale was classified as follows: (i) complete healing from 0 to 2; (ii) moderate healing from 3-5; (iii) mildly healed from 6-8; and (iv) unhealed from 9 to 15.

Tool III: Modified Oxford Scale (MOS)

Newman and Laycock [11] created the MOS to evaluate the strength of the pelvic floor muscles by vaginal examination. The scale has six points: 0 for no contraction, 1 for flicker, 2 for weak, 3 for moderate, 4 for good (with lift), and 5 for strong.

Pain and wound integrity were assessed in research participants immediately after delivery, on postnatal day 2, in the second week, and finally in the sixth week postpartum. The tone of the perineal muscles was assessed at the second and sixth weeks postpartum. To eliminate bias, the patient received the VAS before the session began. To eliminate bias, the same observer assessed wound integrity and perineal muscle tone. In our research, a 2 ml injection of 2% lignocaine dissolved in 8 ml of normal saline was used as a local anesthetic prior to episiotomy. All patients had medial-lateral episiotomies, and all of them were sutured with 2-0 fast vicryl using the interrupted suturing method after all aseptic measures had been taken.

Statistical analysis

The quantitative variables, means, and standard deviations were calculated for the descriptive analysis. We computed absolute frequencies and quartiles for categorical variables. An independent two-sample t-test and a chi-square test were used to examine the relationship between the variables. Statistical significance was determined for all tests with p-values < 0.05. This statistical study was performed using IBM SPSS Statistics (version 22.0).

## Results

In this study, we enrolled 208 participants who were evenly divided into two groups: FIR therapy (n = 104) and SB therapy (n = 104). The demographic and clinical characteristics of the participants were closely matched between the two groups (Table 1). The mean age group among the FIR group participants was 23.87 ± 2.99 years, and the SB group was 24.09 ± 3.29 years. The majority of the participants in both FIR and SB therapy were between 21 and 25 years old [45.2% (n=47) and 48.1% (n=50)]. Body mass index (BMI) distributions were also comparable, as 66.3% (n=69) of FIR and 72.1% (n=75) of SB participants had a BMI of 25-29.9 kg/m^2^. The duration of the second stage of labor was predominantly 30-60 minutes in both groups [65.4% (n=68) in FIR vs. 63.5% (n=66) in SB]. The majority of newborns had a birth weight between 2.5 and 3.5 kg [76% (n=79) in FIR vs. 71.2% (n=74) in SB].

**Table 1:** The distribution of the participants undergoing FIR and SB therapy under multiple clinical parameters FIR: far-infrared; SB: Sitz bath (a p-value of less than 0.05 is considered statistically significant). This table depicts the socio-demographic variables of the participants undergoing FIR and SB therapy and illustrates the significant differences between them.

Factors	Sub-factors	FIR therapy	SB therapy	p-value
		n (%)	n (%)	
Number of participants	-	104 (50)	104 (50)	-
Age (years)	Mean	23.87 ± 2.99	24.09 ± 3.29	0.613 (unpaired t-test)
	<20	20 (19.2)	17 (16.3)	0.717
	21–25	47 (45.2)	50 (48.1)	
	26–30	37 (35.6)	36 (34.3)	
	>30	0 (0)	1 (1)	
Body mass index (kg/m^2^)	18.5–24.9	29 (27.9)	21 (20.2)	0.403
	25–29.9	69 (66.3)	75 (72.1)	
	30–34.9	6 (5.8)	8 (7.6)	
Nutritional status	Moderately built and nourished	98 (94.2)	96 (92.3)	0.580
	Well-built and nourished	6 (5.8)	8 (7.7)	
Gestational age	37–38 weeks + 6 days	40 (38.5)	50 (48.1)	0.539
	39–40 weeks + 6 days	43 (41.3)	38 (36.5)	
	41 weeks + 6 days	14 (13.5)	10 (9.6)	
	>42 weeks	7 (6.7)	6 (5.8)	
Initiation of labor	Spontaneous	65 (62.5)	59 (56.7)	0.397
	Induced	39 (37.5)	45 (43.3)	
Duration of second stage labor (minute)	<30	34 (32.7)	38 (36.5)	0.324
	30–60	68 (65.4)	66 (63.5)	
	>60	2 (1.9)	0 (0)	
Length of episiotomy (cm)	<4	20 (19.2)	26 (25)	0.578
	4–5	73 (70.2)	69 (66.3)	
	>5	11 (10.6)	9 (8.6)	
Birth weight (kg)	<2.5	10 (9.6)	13 (12.5)	0.712
	2.5–3.5	79 (76)	74 (71.2)	
	>3.5	15 (14.4)	17 (16.3)	

**Figure 2:**
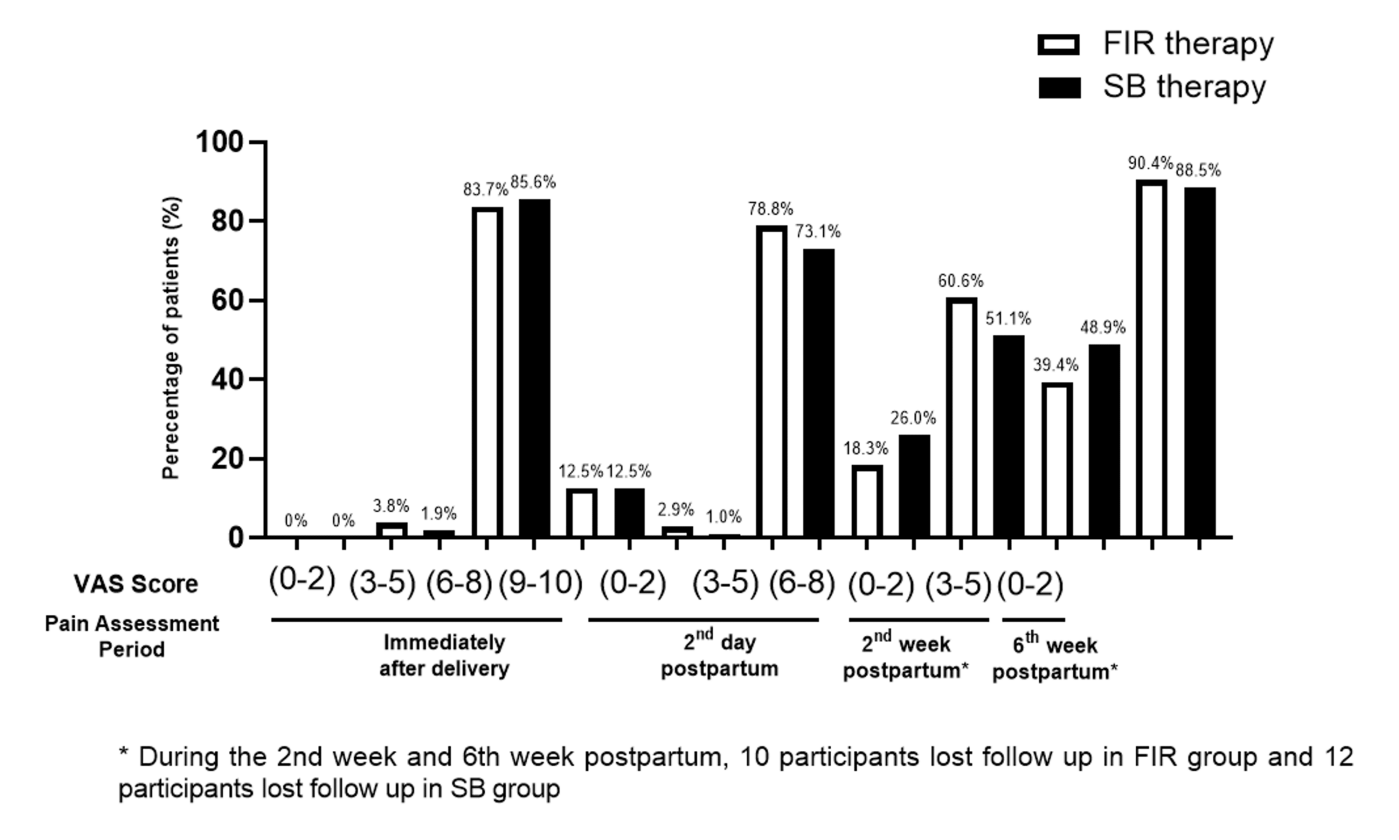
The assessment of pain after FIR and SB therapy This figure depicts the assessment of pain of participants undergoing FIR and SB therapy in four different sessions using VAS scale, i.e., immediately after delivery, post natal day 2, at second week and lastly, at sixth week postpartum.

Pain assessment was conducted at various periods using the Visual Analog Scale (VAS) (Table 2) (Figure 2). Immediately after delivery, the majority of participants in both the FIR therapy and SB therapy groups reported moderate to severe pain (VAS score 6-8), with 83.7% (n=87) in the FIR group and 85.6% (n=89) in the SB group. A smaller proportion reported very severe pain (VAS score 9-10), with 12.5% (n=13) in both groups. On the second day postpartum, the distribution of pain scores indicated that most participants experienced mild pain (VAS score 3-5), with 78.8% (n=82) in the FIR group and 73.1% (n=76) in the SB group. At the sixth week postpartum, a significant majority of participants in both groups reported minimal to no pain (VAS score 0-2), with 90.4% (n=94) in the FIR group and 88.5% (n=92) in the SB group, indicating substantial pain relief over time.

**Table 2:** The assessment of pain after FIR and SB therapy *Ten participants lost follow-up in the FIR group and 12 in the SB group in the second and sixth weeks of postpartum. VAS: Visual Analog Scale; FIR: far-infrared; SB: Sitz bath (a p-value of less than 0.05 is considered statistically significant). This table depicts the assessment of pain of participants undergoing FIR and SB therapy in four different sessions using the VAS scale, i.e., immediately after delivery, postnatal day 2, at the second week, and lastly, at the sixth week postpartum.

Pain assessment period	VAS score	FIR therapy	SB therapy	p-value
		n (%)	n (%)	
Immediately after delivery	3–5	4 (3.8)	2 (1.9)	0.708
	6–8	87 (83.7)	89 (85.6)	
	9–10	13 (12.5)	13 (12.5)	
2^nd^ day postpartum	0–2	3 (2.9)	1 (1)	0.270
	3–5	82 (78.8)	76 (73.1)	
	6–8	19 (18.3)	27 (26)	
2^nd^ week postpartum*	0–2	57 (60.6)	47 (51.1)	0.190
	3–5	37 (39.4)	45 (48.9)	
6^th^ week postpartum*	0–2	94 (90.4)	92 (88.5)	-

**Figure 3:**
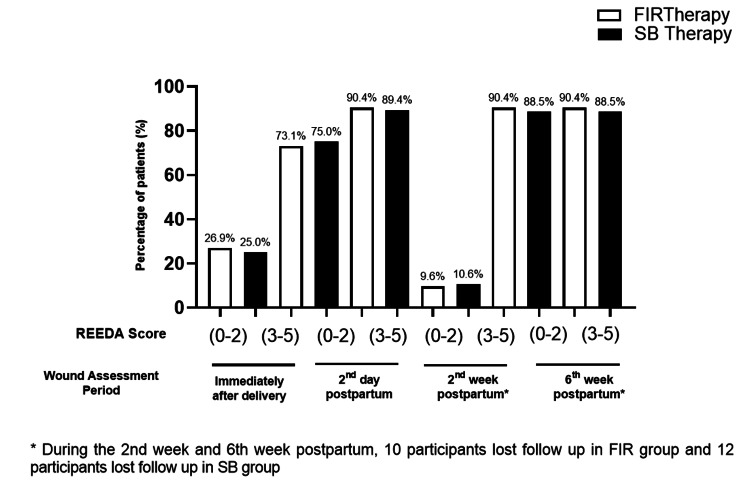
The assessment of wound after FIR and SB therapy This figure depicts the assessment of wound of participants undergoing FIR and SB therapy in four different sessions using REEDA scale, i.e., immediately after delivery, post natal day 2, at the second week and lastly, at the sixth week postpartum.

The wound integrity of participants was evaluated at various intervals using the REEDA score, comparing the effects of FIR therapy and SB therapy (Table 3 and Figure 3). Immediately after delivery in the FIR group, 26.9% (n=28) of participants had a REEDA score of 0-2, indicating better wound integrity, compared to 25% (n=26) in the SB group. Most participants in both groups had REEDA scores of 3-5, with 73.1% (n=76) in the FIR group and 75% in the SB group, showing no significant difference (p = 0.752). By the second week postpartum, the proportion of participants with REEDA scores of 0-2 remained high, with 90.4% (n=94) in the FIR group and 88.5% (n=92) in the SB group. At the sixth week postpartum, the wound integrity continued to show excellent outcomes in both groups, with 90.4% (n=94) of the FIR group and 88.5% (n=92) of the SB group having REEDA scores between 0 and 2.

**Table 3:** The assessment of wound integrity after FIR and SB therapy *10 participants lost follow-up in the FIR group and 12 participants in the SB group in the second and sixth weeks of postpartum. REEDA: Redness, Oedema, Ecchymosis, Discharge, and Approximation Scale; FIR: Far-Infrared SB: Sitz bath (a p-value of less than 0.05 is considered statistically significant). This table depicts the assessment of wounds of participants undergoing FIR and SB therapy in four different sessions using the REEDA scale, i.e., immediately after delivery, postnatal day 2, at the second week, and lastly, at the sixth week postpartum.

Wound integrity assessment period	REEDA score	FIR therapy	SB therapy	p-value
		n (%)	n (%)	
Immediately after delivery	0–2	28 (26.9)	26 (25)	0.752
	3–5	76 (73.1)	78 (75)	
2^nd^ day postpartum	0–2	94 (90.4)	93 (89.4)	0.818
	3–5	10 (9.6)	11 (10.6)	
2^nd^ week postpartum*	0–2	94 (90.4)	92 (88.5)	-
6^th^ week postpartum*	0–2	94 (90.4)	92 (88.5)	-

**Figure 4:**
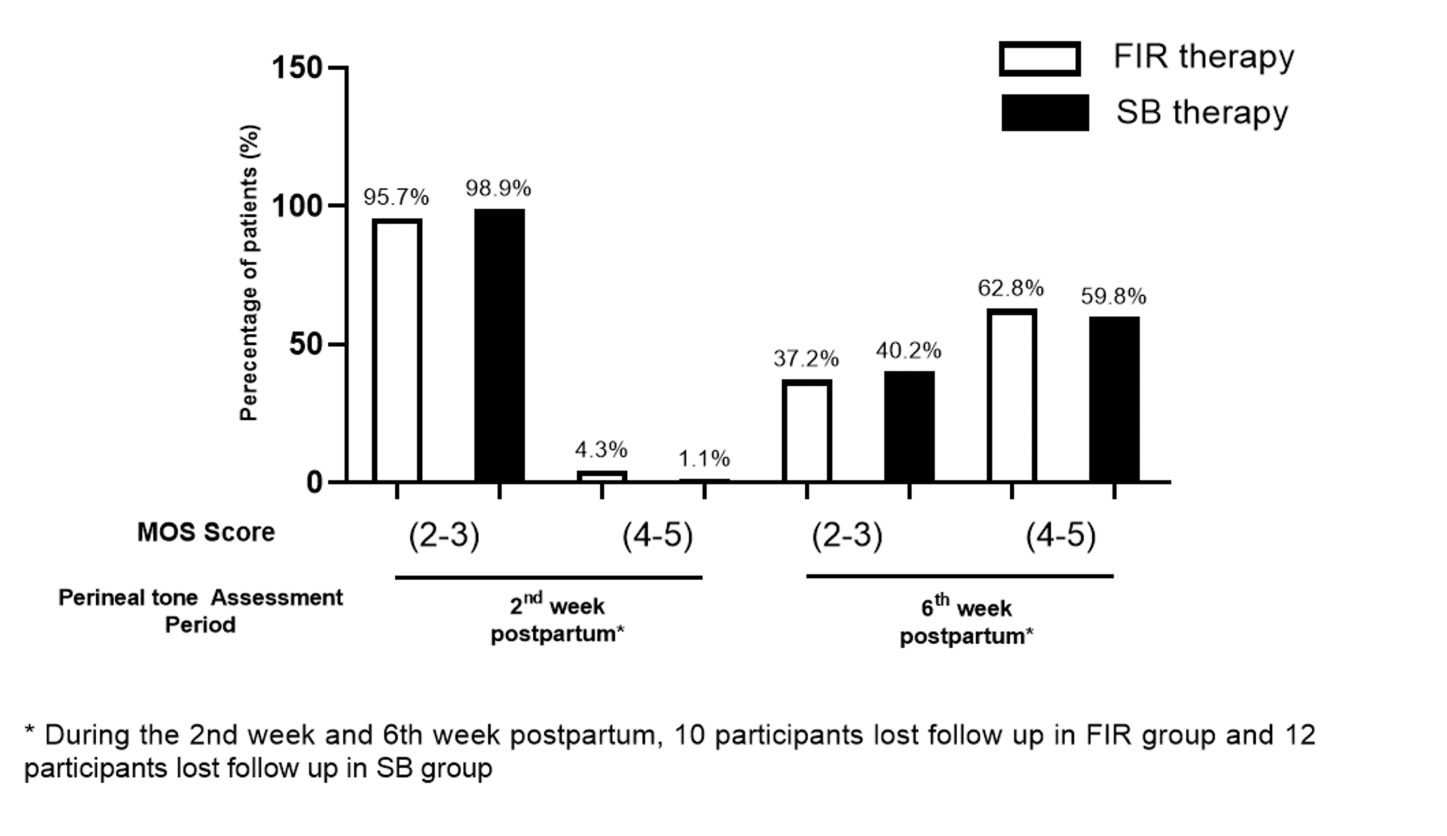
The assessment of perineal tone after FIR and SB therapy This figure depicts the assessment of wound of participants undergoing FIR and SB therapy in two different sessions using MOS scale, i.e., at the second week and at the sixth week postpartum.

The assessment of perineal muscle tone at various postpartum periods was compared between the FIR therapy and SB therapy groups using the MOS scale (Table 4; Figure 4). In the second week postpartum, the majority of participants in both groups had MOS scores of 2-3, with 95.7% (n=90) in the FIR group and 98.9% (n=91) in the SB group. By the sixth week postpartum, the distribution of MOS scores showed a shift towards higher scores. However, the difference was not statistically significant (p = 0.676).

**Table 4:** The assessment of perineal muscle tone after FIR and SB therapy *10 participants lost follow-up in the FIR group and 12 participants in the SB group in the second and sixth weeks of postpartum. MOS: Modified Oxford scale. FIR: far-infrared; SB: Sitz bath (a p-value of less than 0.05 is considered statistically significant). This table depicts the assessment of wounds of participants undergoing FIR and SB therapy in two different sessions using the MOS scale, i.e., at the second week and at the sixth week postpartum.

Perineal muscle tone assessment period	MOS	FIR therapy	SB therapy	p-value
		n (%)	n (%)	
2^nd^ week postpartum*	2-3	90 (95.7)	91 (98.9)	0.182
	4-5	4 (4.3)	1 (1.1)	
6^th^ week postpartum*	2-3	35 (37.2)	37 (40.2)	0.676
	4-5	59 (62.8)	55 (59.8)	

**Figure 5:**
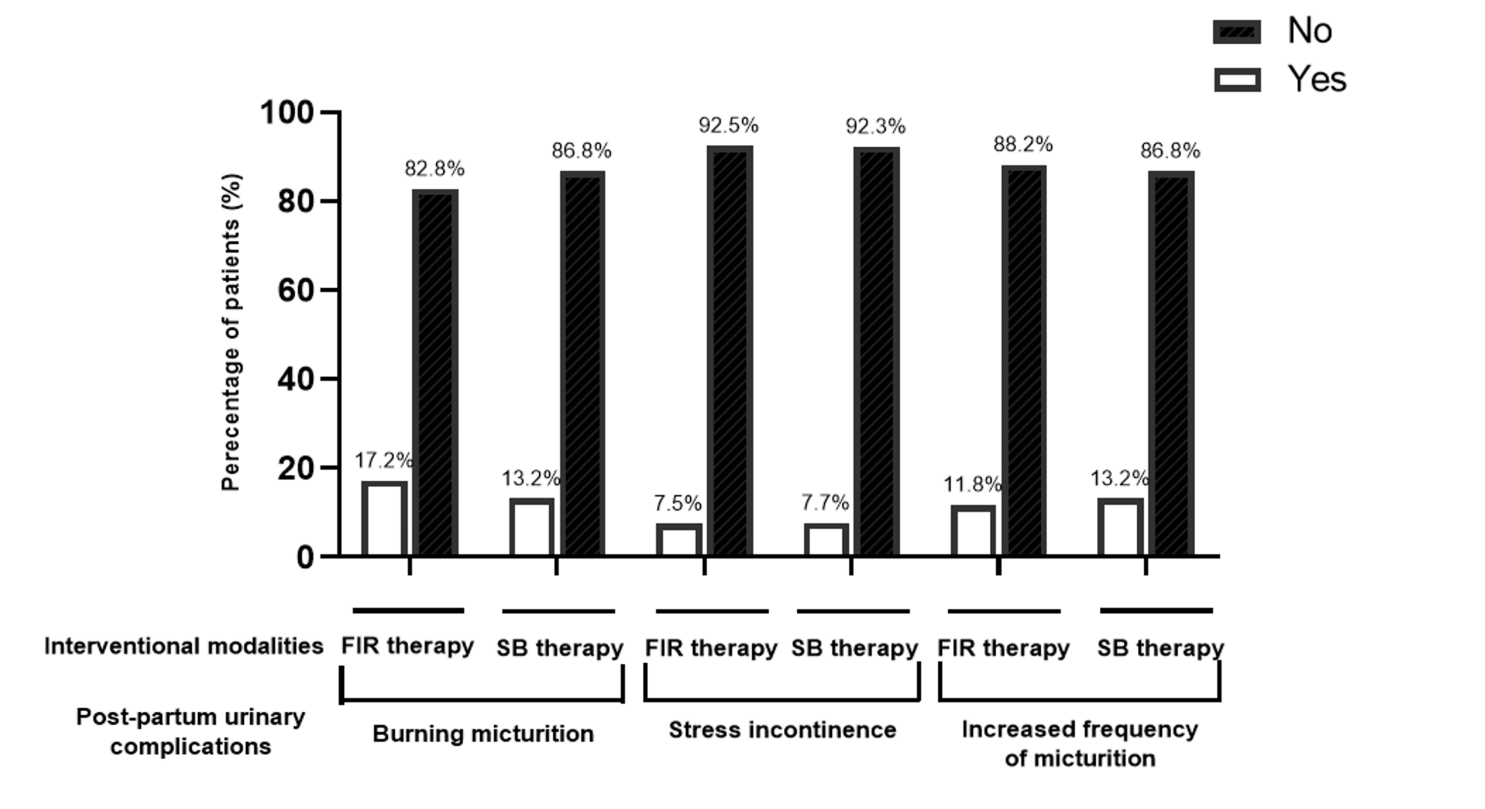
The assessment of post-partum urinary complications after FIR and SB therapy This figure depicts the assessment of post-partum urinary complications of patients undergoing FIR and SB therapy.

The comparison of post-partum urinary complications between the FIR therapy and SB therapy groups was also reported in our study (Table 5 and Figure 5). For burning micturition, 17.2% (n=16) of participants in the FIR group reported experiencing this complication, compared to 13.2% (n=13) in the SB group. This difference was not statistically significant (p = 0.448). Most participants in both groups did not report increased frequency of micturition, with 88.2% (n=82) in the FIR group and 86.8% (n=79) in the SB group.

**Table 5:** Table depicting the post-partum urinary complications after FIR and SB therapy *10 participants lost follow-up in the FIR group and 12 participants in the SB group at the second and sixth weeks of postpartum; hence, postpartum urinary complications were seen among 94 participants in the FIR therapy group and 92 participants in the SB therapy group. FIR: far-infrared; SB: Sitz bath (a p-value of less than 0.05 is considered statistically significant). This table depicts the assessment of post-partum urinary complications in patients undergoing FIR and SB therapy.

Post-partum urinary complications*	Yes/No	FIR therapy	SB therapy	p-value
		n (%)	n (%)	
Burning micturition	Yes	16 (17.2)	13 (13.2)	0.448
	No	78 (82.8)	79 (86.8)	
Stress incontinence	Yes	7 (7.5)	8 (7.7)	0.966
	No	87 (92.5)	84 (92.3)	
Increased frequency of micturition	Yes	12 (11.8)	13 (13.2)	0.781
	No	82 (88.2)	79 (86.8)	

## Discussion

All of the patients in our study were primiparous women, which was comparable to the studies done by Nethravathi et al. [12] and Huang et al. [13]. However, other studies conducted by Ismail and Ghatta [14], Chandraleka et al. [15], and Eko et al. [16] included both primiparous and multiparous women. Multiparous women already have compromised perineal tone; thus, excluding them from the study can be considered a better option for a clearer and more unbiased comparison between FIR and SB on perineal muscle tone after delivery. The mean age of the primiparous women in the FIR and SB groups in our study was 23.87 ± 2.99 years and 24.09 ± 3.29 years, which was comparable to studies done by Ismail and Ghatta [14], Eko et al. [16], and Kaur et al. [17]. The average length of episiotomy in our study ranged from 4 to 5 cm, which was slightly higher than that of other studies. For example, both Jahdie et al. [18] and Kaur et al. [19] had an average episiotomy length of 3-4 cm. The average birth weight in our study was between 2.5 and 3.5 kg in both the FIR and SB groups, comparable to studies conducted by Huang et al. [13] and Kaur et al. [19].

Our study found that the FIR group had quicker perineal pain alleviation than the SB group from birth to the second day and second week. However, the difference was not statistically significant. We measured participants' perineal pain using VAS. Huang et al. [13] and Eko et al. [16] employed a similar strategy. However, Chandraleka et al. [15] assessed perineal pain using a universal pain scale, and Ismail and Ghatta [14] utilized a numerical pain rating scale. REEDA is the standard wound integrity evaluation instrument used in several studies [12,14,15,17] to evaluate episiotomy approximation. Girsang and Elfira [20] assessed wound healing using Southampton and Bates-Jensen wound assessment ratings. Our investigation found that FIR improved wound integrity more than SB, although the difference was not statistically significant. Nethravathi et al. [12] and Kaur et al. [19] found that FIR light treatment heals postpartum episiotomy wounds. Our study employed the same suture material and aseptic approach as Kaur et al. [17]. Unlike our study, they found SB therapy had a far higher success rate. In our research, the FIR group improved perineal muscle tone somewhat more than the SB group, but not significantly. Handa et al. [21] showed that six weeks postpartum, 16% (n=71) of patients had stress incontinence, 10% (n=45) had hyperactive bladders, and 12% (n=56) had anal incontinence after episiotomy/perineal laceration. Forceps deliveries and perineal lacerations caused pelvic floor anomalies 5-10 years after the first delivery, unlike episiotomies.

One of the strengths of our research is that we included only primiparous women. Multiparous women are known to have compromised perineal muscle tone, so enrolling only primiparous women undergoing episiotomies reduces the selection bias. Also, as our study is conducted by a single observer, the bias is further reduced. Our study also has a few limitations. First, our sample size was relatively small. A larger sample size could have increased the statistical significance of our study. Second, we did not perform a follow-up study. Following up on the participants for a longer time is required for assessing long-term complications like bladder, bowel, and sexual dysfunction. Lastly, we used VAS for pain assessment, a subjective scale.

## Conclusions

The use of FIR therapy has shown better results in reducing immediate post-delivery pain and improving wound healing and the tone of perineal muscle. FIR therapy has better compliance and patient satisfaction, is more user-friendly and cost-efficient, and requires a one-time investment compared to SB therapy. Our study did not report any statistically significant results; however, more longitudinal studies should be conducted at a wider range to infer the significant effects of the therapies. This will not only contribute to the clinicians' knowledge regarding handling such situations in an efficient way but also help improve postnatal care in different tiers of health setups.
